# Probiotics and vaginal microecology: fact or fancy?

**DOI:** 10.1186/s12905-019-0723-4

**Published:** 2019-01-31

**Authors:** Laura Buggio, Edgardo Somigliana, Alessandra Borghi, Paolo Vercellini

**Affiliations:** 1 Gynaecology Unit, Fondazione IRCCS Ca’ Granda Ospedale Maggiore Policlinico, Via Commenda, 12 - 20122 Milan, Italy; 2 Deparment of Clinical Sciences and Community Health, Università degli Studi, Milan, Italy; 3 Infertility Unit, Fondazione IRCCS Ca’ Granda Ospedale Maggiore Policlinico, Milan, Italy

**Keywords:** Probiotics, Obstetrics, Gynaecology, Preterm birth, Bacterial vaginosis, Vulvovaginal candidiasis, Lactobacilli, Pregnancy

## Abstract

**Background:**

Probiotics are live microorganisms that, when administered in adequate amounts, should confer a health benefit to the host. Media sources tend to present probiotics as an appealing health promotion method able to prevent or treat a wide variety of clinical conditions. In obstetrics and gynaecology, *Lactobacilli* species are mainly used to restore the physiologic vaginal microbiota in order to treat bacterial vaginosis and vulvovaginal candidiasis (VVC) and prevent preterm birth.

**Discussion:**

Several RCTs investigated the potential benefits of probiotics in gynaecological and obstetrics conditions. For all potential indications, recent specific meta-analyses have been published. Considering vulvovaginal candidiasis in non-pregnant women, probiotics slightly improved the short-term clinical and mycological cure, and reduced the 1-month relapse. However, no important impact of probiotic use was observed on long-term clinical or mycological cure. Similarly, the addition of probiotics to metronidazole for the treatment of bacterial vaginosis was not shown to provide any additional benefit. In obstetrics, using probiotics during pregnancy neither decreased nor increased the risk of preterm birth before 34 weeks or before 37 weeks. Similarly, no benefits emerged for gestational diabetes, preterm premature rupture of membrane, and small and large for gestational age infants.

**Conclusion:**

Despite increasing marketing of probiotics for the treatment of vulvovaginal candidiasis and prevention of preterm birth robust evidence demonstrating a beneficial effect is scarce. Moreover, there was considerable heterogeneity among the different studies in terms of route of administration, strain/s of probiotic adopted, and length of probiotic use. Before recommending the systematic use of probiotics to treat bacterial vaginosis and VVC and prevent preterm birth, high-quality research is needed. Professional medical associations should issue recommendations defining if, when, and how probiotics should be used for gynaecological disorders.

## Background

According to the World Health Organization (WHO), probiotics are live microorganisms that, when administered in adequate amounts, confer a health benefit to the host [1]. Probiotics can be ingested with diet or in supplement forms [2]. Their consumption has been proven effective for the management of some gastrointestinal conditions, such as irritable bowel syndrome, and for the prevention of diarrhoea associated with *Clostridium difficile* infection [3, 4].

In the last decade, many clinical trials have been conducted to assess the effects of probiotics in the prevention and treatment of a broad range of disorders, and the scientific interest in this field is growing. Searching Medline through PubMed for “probiotics”, identifies 14.188 articles published between 2007 and 2017 (accessed 14 January 2018), with an increase of 163% from 2007 to 2017 in the number of articles published per year. In the obstetrical and gynaecological field probiotics, administered both orally and vaginally, have been mainly tested for the prevention and treatment of vaginal infections and for the prevention of preterm birth [2, 5–8]. The rationale of using oral probiotics in the treatment of gynaecological conditions is related to the ability of these microorganisms to survive through the gastrointestinal system and to ascend to the vaginal tract after their excretion from the rectum; whereas vaginal administration allows a direct and targeted colonization action of the probiotics for restoring unhealthy vaginal microbiota [9].

Also, the use of probiotics is progressively expanding. In particular, women of reproductive age are prone to use these products for gastrointestinal symptoms [10]. Another important catchment area is represented by pregnant women, as it is estimated that up to 1 woman out of 7 in the Netherlands regularly use probiotics during gestation [11].

Media sources tend to present probiotics as an appealing health promotion method able to prevent or treat a wide variety of clinical conditions [12]. Indeed, in 2015 probiotics market exceeded $35 billion and it is estimated to continue to rise in the years to come [13]. An estimate based on a survey conducted for FederSalus (the main Italian referent for institutional and commercial organizations operating in the field of food supplements) on more than 6000 individuals representing the Italian population aged 18 and above, indicated that 32 million Italians have used a nutritional supplement in the last year [14]. In Italy, from November 2016 to October 2017 the market of nutritional supplements reached 2.9 € billions, for a total of almost 212 million packs sold, with an increase in turnover of 7.3% [15]. In particular, in Italy the probiotic market reached € 343 million in 2016, ranking first among the best-selling food supplements [16]. In other Western countries the scenario is similar. In fact, in the North American market (United States and Canada) probiotic represent the category of nutritional supplement with the higher growth in absolute terms (+$725 million) in the period 2009–2014 [17].

Given this background, the time has come to verify whether probiotics really benefits pregnant and non-pregnant women, and whether the magnitude of the effect justifies the expenditure.

## Probiotics in non-pregnant women

Vaginal infections represent one of the most common reason for gynaecological consultation [7]. It is estimated that approximately seven women out of 10 will experience at least one episode of vulvovaginal candidiasis (VVC) in their lives [18]. Bacterial vaginosis (BV) is another highly prevalent vaginal disorder associated with an increased risk for pelvic inflammatory disease, sexually transmitted infections, HIV transmission, and preterm delivery [19]. Bacterial vaginosis is characterized by a reduction or depletion of lactobacilli and overgrowth of *Gardnerella vaginalis, Mycoplasma hominis*, *Prevotella* species, and other pathogenic anaerobic bacteria [18]. *Lactobacillus* species produce lactic and acetic acid and hydrogen peroxide, maintain the vaginal pH around 4.5 or less, hamper growth of pathogenic bacteria and *Candida albicans*, and are thus considered protective against VVC and BV [19, 20]. Accordingly, the putative beneficial effect of *Lactobacillus* species-containing probiotics in restoring and maintaining the physiologic vaginal microbiota, fostered their use for the treatment of both vaginal disorders.

The effectiveness of probiotics for the treatment of VVC in non-pregnant women was recently evaluated in a Cochrane systematic review [7]. A total of 10 RCTs (1656 participants) investigating the effect of probiotics used by the oral and vaginal route as a complementary therapy to conventional antifungal drugs were included. Probiotics slightly improved the short-term clinical and mycological cure rate (risk ratio (RR) 1.14, 95% CI 1.05–1.24, and RR 1.06, 95% CI 1.02–1.10 respectively), and reduced the 1-month relapse rate (RR 0.34, 95% CI 0.17–0.68). However, no important impact of probiotic use was observed on long-term clinical or mycological cure rate (3-month post-treatment evaluation, RR 1.30, 95% CI 1.00–1.70; and RR 1.16, 95% CI 1.00–1.35, respectively). Given the low- or very low-quality of the considered studies, the authors emphasized the need for further and better designed RCTs with larger sample size, standardized methodology for probiotic preparation, and longer follow-up, in order to define also other outcomes that may matter to women, such as time to first relapse, need for repeated or prolonged treatments, patient satisfaction, and cost effectiveness. However, we have to underline that one of the major and unresolved issues related to the treatment of VVC is the high rate of recurrences even after the use of conventional azoles treatment [21, 22]. In addition, treatment of recurrent VVC, defined as four or more symptomatic episodes within 12 months, could be challenging due to the increased presence of azole-drug resistance [23]. Moreover, in complicated forms, all treatments, including antimycotics, are not supportive of long-term beneficial results. In this particular sub-group of patients, the protective role of specific *Lactobacillus* species-containing probiotics, such as *Lactobacillus plantarum* P17630, has been proven effective as a potential empirical preventive agent of VVC recurrences [23].

The putative beneficial effect of probiotics supplementation for the treatment of BV has been assessed in various meta-analysis [8, 24, 25]. A 2009 Cochrane review [24] showed promising results derived from the use of oral and vaginal probiotics combined with metronidazole or used alone. In 2013, a systematic review [25] supported the potential beneficial effect of probiotics for the treatment of BV. Huang et al. [25] included in their analysis twelve RCTs published between 1992 and 2012; probiotics were adopted either orally (*n* = 8) or vaginally (*n* = 4), with follow-up periods ranging from 4 weeks to 6 months. The pooled result showed that probiotics supplementation was able to significantly improve the cure rate in adult BV patients (RR 1.53, 95% CI 1.19–1.97). Subgroup analyses failed to demonstrate a beneficial effect of probiotics supplementation in terms of long-term (> 1 month) follow-up (RR 1.15, 95% CI 0.89–1.47), and a substantial heterogeneity was shown across different study designs. Finally, in a recent meta-analysis [8], the authors compared the use of metronidazole alone with the combination of this antibiotic plus probiotics. Five RCTs including a total of 1186 participants were selected. An overall risk ratio of 0.98 (95% CI 0.91–1.06; *P* = 0.57) was observed for the cure rate achieved with combined therapy over metronidazole alone on BV.

At now, given the presence of inconclusive results, some international guidelines do not support the use of probiotics for the treatment of vulvovaginal infections (Table 1).

**Table 1:** Role of probiotics in International guidelines for the treatment of vulvovaginal infections

Guideline	Role of probiotics
European (IUSTI/WHO) guideline, 2011 [39]	Potential role of vaginal probiotics in the management of recurrent BV
Faculty of Sexual & Reproductive Healthcare, Royal College of Obstetricians and Gynaecologists (RCOG), 2012 [40]	*Recurrent BV*: There is currently insufficient evidence to recommend the use of probiotics either before, during or after antibiotic treatment as a means of reducing recurrence. *Recurrent VVC*: Non-conventional management regimens such as dietary changes, use of probiotics, tea tree oil and not wearing tight clothing have been studied. There is currently insufficient evidence to support their recommendation in treatment.
German Society for Gynecology and Obstetrics, 2015 [41]	Probiotics have shown encouraging, but controversial results and require further investigation
Society of Obstetrician and Gynaecologyst of Canada (SOGC), 2015 [42]	Current evidence of the efficacy of alternative therapies for bacterial vaginosis (probiotics, vitamin C) is limited (I).
Centers for Disease Control and Prevention (CDC), 2015 [43]	Overall, no studies support the addition of any available lactobacillus formulations or probiotic as an adjunctive or replacement therapy in women with BV. Further research efforts to determine the role of these regimens in BV treatment and prevention are ongoing

*IUSTI/WHO* = International Union against Sexually Transmitted Infection and the World Health Organization

*BV* = bacterial vaginosis

*VVC* = vulvovaginal candidiasis

## Probiotics in pregnant women

It has been suggested that probiotics could play a role in the prevention of preterm birth [2, 6]. Preterm birth rates vary across different countries, ranging from 5 to 9% in Europe to 13% in USA [26]. The aetiology of preterm birth is multifactorial, but it has been estimated that about one third of cases is due to intrauterine inflammation [26] caused by ascending vaginal infections. In particular, a pre-existing BV appears to be strongly associated with premature birth [5].

Therefore, here the putative role of probiotics might be associated with their potential ability to displace and kill pathogens. The hypothesized mechanisms include the development of anti-inflammatory cytokines and the reduction of the vaginal pH, so that the vaginal environment becomes again favourable to the growth of healthy bacteria [6, 20]. Moreover, the use of probiotics in pregnancy could improve maternal glucidic metabolism through the modification of gut microbial composition and function, as well as the improvement of insulin sensitivity [27].

To verify these hypotheses, Jarde et al. [2] performed a systematic review and meta-analysis on the risk of preterm birth and other adverse pregnancy outcomes in women with a singleton pregnancy receiving probiotics. In this analysis the authors included also prebiotics, i.e. food ingredients that indirectly induce the growth or activity of beneficial microorganisms. A total of 21 studies (4098 women) were included in the final analysis. Five studies (1017 women) evaluated the risk of preterm birth < 34 weeks of gestation, whereas the risk < 37 weeks was assessed in 11 studies (2484 women). Using probiotics during pregnancy neither decreased nor increased the risk of preterm birth before 34 weeks (RR 1.03; 95% CI:0.29–3.64) or before 37 weeks (RR 1.08; 95% CI: 0.71–1.63). In addition, the authors did not observe a protective effect of probiotics supplementation on most of the secondary outcomes considered, including gestational diabetes, preterm premature rupture of membrane (PPROM), and small and large for gestational age infants. The only statistically significant difference in favour of probiotics supplementation regarded glucose metabolism (Homeostatic Model Assessment of Insulin Resistance (HOMA-IR) and Insulin); however, the pooled estimate on gestational diabetes did not show any benefit from probiotics intake (RR 1.25; 95% CI 0.61–2.56).

In contrast with these results, a Greek RCT [28] showed a benefit of probiotic administration in women with PPROM. Patients were allocated to 10-days vaginal probiotic supplementation in combination with antibiotic prophylaxis (*n* = 59) or to standard antibiotic treatment alone (*n* = 57). In women treated with the double regimen significantly higher mean gestational age at birth (35.49 vs 32.53 weeks) and latency period (5.60 vs 2.48 weeks) were observed in comparison to control group.

A recent Norwegian population based-prospective cohort study [29] investigated the potential association between the consumption of probiotic milk and the incidence of preterm delivery and preeclampsia. Maternal inflammatory response represents the common background of these two pathologic conditions, and the potential anti-inflammatory effect of probiotics represents the criterion for their use [30, 31]. The authors showed that probiotic milk consumption during late pregnancy, but not before or in early pregnancy, was associated with a reduced risk of preeclampsia (adjusted OR: 0.80; 95% CI, 0.64-0.94). Regarding preterm birth, ingestion of probiotic milk during early pregnancy, but not before or in late pregnancy, was associated with a reduction in risk of preterm delivery (adjusted OR: 0.79; 95% CI, 0.64-0.97). In both cases, no dose-response relationship was found.

The putative role of oral probiotics on vaginal micro-environment in pregnancy has been evaluated by Gille et al. [32] in a randomized, triple-blind, controlled trial (RCT) conducted on 320 women. Participants were allocated to oral probiotic supplementation or placebo. The primary study outcome was the proportion of swabs with normal Nugent score (< 4) after eight weeks of treatment. Oral probiotics did not increase the proportion of normal vaginal microbiota compared to placebo. At post-intervention analysis, the proportion of normal vaginal microbiota decreased from 82.6 to 77.8% in the probiotic group, and from 79.1 to 74.3% in the placebo group, without significant between-group difference (*P* = 0.29).

An Australian double-blind RCT [33] assessed the impact of oral probiotics on vaginal Group B Streptococcal (GBS) colonization rates in 34 women. Only women with a GBS-positive vaginal swab at 36 weeks were deemed eligible for the study. Patients were assigned to daily oral probiotics plus standard antenatal care (intervention group) or standard antenatal care (control group) for three weeks or until delivery. At the end of the treatment period, no significant between-group difference was observed in GBS infection rate.

Finally, the results of a recent systematic review [34] do not support the treatment of BV-positive pregnant women with probiotics with the objective of reducing the risk of spontaneous preterm delivery.

## Probiotics in obstetrics and gynaecology: patient’s health or industry wealth?

From a commercial point of view, it appears that the “golden era” of probiotics has begun. In the words of Arnold [35] “like all good bacteria, probiotics have sprung forth and multiplied. Trillions live in our guts, and even more have begun to occupy grocery store shelves”. However, to be defined a “probiotic”, the strain of bacteria must have demonstrated health benefits [35].

The theoretical benefits deriving from the increase in the number of healthy vaginal bacteria at the expenses of potentially pathogenic micro-organisms appears intuitive. However, in many cases it is unclear whether the alteration of the vaginal microbiota is a consequence of incidental infections or of a systemic endocrine/immunologic/metabolic condition predisposing to lactobacillus extinction. In the former case, re-introducing physiologic bacteria after pharmacological eradication of pathogens seems rational, but in the latter one it would result in trying to cure the consequence rather than the origin of the disorder. The high BV and VVC relapse rate observed after both antibiotic treatment and complementary probiotic use suggests that the second hypothesis might be true. If this is the case, probiotics might reveal no more than an incomplete, temporary, and expensive remedy. Another practical issue is the route of administration. In fact, whether intravaginal insertion of probiotics seems logical [19], aiming at modifying the vaginal microbiota via oral ingestion of physiologic bacteria appears less intuitive, and implies ingested probiotics to reach the rectum, ascend the vagina, and dislodge bacterial pathogens and yeasts [20].

Disappointingly, the widespread use of probiotics to reduce the risk of preterm delivery and to improve the cure rate of BV and VVC does not seem to be justified by the currently available data. In fact, despite increasing marketing and sales of probiotics, the results originated by clinical trials are inconsistent and generally of sub-optimal quality. Moreover, a substantial proportion of these trials have been sponsored by parties with a commercial interest in the outcome [36]. In addition, there was considerable heterogeneity in published studies in term of strain/s of probiotic adopted, route of administration (oral, vaginal), and duration of treatment [36].

The effects of probiotics seem to be strain-specific and dose-dependent, and the lack of a standardized manufacturing process could affect microbial survival, growth, and viability [37]. Along this line, a recent position paper by The European Society for Paediatric Gastroenterology Hepatology and Nutrition (ESPGHAN) Working Group provides evidence on the inadequate quality of commercial probiotic products, in terms of definition of microorganisms, their numbers, functional properties, and presence of contaminating microorganisms. The Working Group suggests the creation of certified laboratories where the quality control of probiotics should be performed using universally shared validated and standardized methodologies [37]. In addition, as it is the case with drugs, adverse events potentially related to the use of probiotics, should be reported to and registered by health authorities [37].

The regulatory aspects related with the production and marketing of these products constitute another reason of concern for both patients/consumers and physicians. The regulation of probiotics differs between countries without a universally shared framework [38]. In general, probiotic products are classified as food or dietary supplements, and their development process has to fulfil considerably less rigorous regulatory criteria compared with drugs [37]. However, if probiotics are to be prescribed to patients with specific disorders, they should be regulated as drugs rather than foods or supplements. Nevertheless, we are not aware of any indication to probiotic use in obstetrics and gynaecology approved by national and supranational regulatory agencies such as Food and Drug Administration (FDA) and European Medical Agency (EMA).

Indeed, the somewhat vague manufacturers claims may generate a sort of nobody’s land where commercial interests may flourish independently of the effect of probiotics for the prevention and treatment of specific disorders [35]. In fact, as in most countries the regulation of probiotics is focused on the legitimacy of any claim rather than on their efficacy, manufacturers are careful not to mention definite medical indications for their products [38]. In this regard, professional medical associations should issue recommendations concerning the role of probiotics in obstetrics and gynaecology, as their uncontrolled implementation might also lead to a potentially harmful decrease in the use of effective standard drug treatments.

The primary aim of probiotics is the re-establishment of a physiological vaginal microbiome. However, there is currently no consensus regarding their use for the treatment of vaginal infections and their sequelae. Thus, further better-quality data are needed to define the real effect size of probiotic use in different obstetrical and gynaecological conditions. At the very least, our duty is to provide complete and quantitative information to patients/consumers, allowing them to decide whether probiotics are worth their cost.

## Abbreviations

BV: Bacterial vaginosis
EMA: European Medical Agency
FDA: Food and Drug Administration
GBS: Group B Streptococcal
HOMA-IR: Homeostatic Model Assessment of Insulin Resistance
PPROM: Preterm premature rupture of membranes
RCT: Randomized controlled trial
RR: Risk ratio
VVC: Vulvovaginal candidiasis
WHO: World Health Organization
